# Gleanings

**Published:** 1893-11

**Authors:** F. H. Lutze

**Affiliations:** Brooklyn, N. Y.


### GLEANINGS.

F. H. Lutze, M. D., Brooklyn, N. Y.

### Teeth and Gums.

Pain in teeth, as if pulled out. Arn., Caust., Nux-mosch., Nux-vom., Phos-ac., Rhus-t., Ipec., Cocaine, Cyclam., Mangan., Mezer.

Teeth decay down to gums. Calc-c.

— — root is affected only. Thuja.

— — crown is affected only. Staphys.

Canker sores in mouth, burning; all food and drink aggra vates. Sinapis-alb.

Tongue bluish white on upper and lower surface. Glon.

Dryness of the mouth, intense, without thirst. Paris-quad., Dioscor-vil., Lycopod., Nux-mosch., Puls., Sulph.

Hair on forepart of tongue, sensation of. Silicea, Apis.

— — base — —. Kali-bich.

Teething in children, crying, restless, feverish, biting on something hard, and > by it, < at night. Phytolacca.

Palate and uvula bright red, uvula looks like a bag of water. Kali-bich. (Apis, Rhus-t., Phos., Sulph.-ac., Tabac.)

Tongue indented on edge with marks of teeth. Glon., Iod., Rhus, Hydrast., Ignat., Kali-iod., Podoph., Stram., Tellur.

Tongue has a triangular red tip. Argent-n., Ars., Phytol., Rhus, Sulph.

Tongue swollen, seems to fill up the whole mouth. Cajeput.

Tongue catches on teeth. Apis.

Tongue protruded and retracted like a snake. Lachesis (Cupr-acet.).

— slick and shining. Kali-bich.

Tongue excoriated, patches like islands on the tongue, craving for fat. Merc-viv.

Mouth bleeding from buccal cavity in diphtheria. Phytolacca.

Mouth and tongue dry and burning, constant thirst for large quantities of ice cold water, which is vomited so soon as it becomes warm in stomach, gurgling from stomach down through abdomen, causing an involuntary stool from relaxed anus. Phos.

### Fauces, Pharynx, and Œsophagus.

*Throat*, if a fishbone has wounded the œsophagus. Cicuta-vir.

— — — — — — — and a sensation of the fishbone remains but cannot be discovered. Hepar.

Globus hystericus. Acon., Con., Magn-m., Plumbum.

— — > eructations. Magn-m.

Lump in throat descends on deglutition, but immediately returns. Rumex-crisp.

— — — does not move from deglutition. Ignat.

— — — or œsophagus. Asafœt., Con., Laches., Lycopod., Lobel., Magn-c., Plumb., Physostig., Kalmia.

—, swallowing over a lump in throat. Graph., Nat-m., Puls., Sabin.

Splinter, sensation of in throat. Alumina.

Constriction, spasmodic, of throat, interfering with swallowing; food is felt the whole length of the œsophagus; > warm drinks. Alumina.

Throat feels enlarged like a burning cavern. Iris-vers.

— < from least external touch. Bell., Laches., Phos., Rumex., Niccolum.

— extremely dry > drinking. Phos.

— hot feeling in, sputa, in little granules like shot, very offensive when opened. Phos., Silicea.

Clergyman's or speaker's sore throat. Apis, Arum.

Fauces, inflammation of mucous follicles in back part of fauces, like fleshy soft warts or ridges down the throat. Laches.

Throat, chronic sore, beginning on left side. Lachesis, Bell.

Throat, automatic grasping of hands to throat, head, nose, ears, etc. Stramon.

### APPETITE, TASTE, HUNGER, THIRST.

Drinking very cold water or eating ice-cream, pain in forehead extending to nose. Digital.

Eats often but little at a time. Bry.

— — — much — — —. Ars.

Taste of blood in the mouth. Benz-ac., Lil-tig., Alum., Amm-c., Berb., Bell., Bismuth, Bovist., Ferr., Hipp-m., Ipec., Jatroph., Rhus, Sabin., Sil., Zinc.

Vomit, black, in yellow fever. China, Ars., Arn., Crotal., Laches.

— water, when it reaches the stomach; and restlessness. Ars.

— — and all fluids as well, when it reaches the stomach; can eat his rations for several days, then vomits and keeps it up for a whole day. Bismuth.

Regurgitation of food (without nausea). Ammon-m., Asafœt., Con., Croton., Lycopod., Magn-m., Nux-v., Plumb., Ran.-b., Sars., Spig., Verb.

— — — bitter sour. Phos., Podoph., Sabad., > P. M. —.

Blood, vomiting of. Acon., Aloe, Alumina, Amm-c., Argent-nit., Arnic., Arsenic, Bell., Bry., Calc., Canth., Carb-v., Caust., China, Cicuta-vir., Colocynth., Con., Crotal., Cupr-met., Erecthites, Eviger., Ferr-met., Hamam., Hyos., Ipec., Kali-bich., Laches., Lobel-infl., Lycopod., Merc., Millefol., Natr-m., Nux-v., Op., Petrol., Phos., Phytolac., Plumb., Podo., Puls., Rhus-t., Secal., Stan., Stram., Sulph., Veratr-alb., Verat-vir., Zinc.

Vomiting. After vomiting breaks out all over with a profuse perspiration, followed by a sensation as if a thousand needles were piercing skin from within outwards. Lobel-inflata.

Vomit, patient thinks he would be so much better if he could only vomit. Nux-v.

Nausea and vertigo on turning to the left, a fainty sickness. Iod.

Vomiting and purging, with cold, blue, dry skin; during fever and pains in abdomen he covers up; when the skin becomes cold he uncovers. Camph.

Eating, during and after, sleepy. Bovista, Kali-c., Phos., Puls.

